# The Use of Delta Neutrophil Index and Myeloperoxidase Index for Predicting Acute Complicated Appendicitis in Children

**DOI:** 10.1371/journal.pone.0148799

**Published:** 2016-02-09

**Authors:** Oh Hyun Kim, Yong Sung Cha, Sung Oh Hwang, Ji Young Jang, Eun Hee Choi, Hyung Il Kim, KyoungChul Cha, Hyun Kim, Kang Hyun Lee

**Affiliations:** 1 Department of Emergency Medicine, Wonju College of Medicine, Yonsei University, Wonju, Republic of Korea; 2 Department of Surgery, Wonju College of Medicine, Yonsei University, Wonju, Republic of Korea; 3 Biostatistician, Institute of Lifestyle Medicine, Wonju College of Medicine, Yonsei University, Wonju, Republic of Korea; Fondazione IRCCS Ca' Granda Ospedale Maggiore Policlinico, Università degli Studi di Milano, ITALY

## Abstract

**Background:**

In children with acute appendicitis, 30% to 75% present with a complication, such as perforation, and the early diagnosis of complications is known to improve outcomes. Serum delta neutrophil index (DNI) and myeloperoxidase index (MPXI) are new inflammatory markers, and thus, in the present study, the authors evaluated the predictive values of these two markers for the presence of a complication in children with acute appendicitis.

**Methods:**

This retrospective observational study was conducted on 105 consecutive children (<12 years old) with acute appendicitis treated over a 31-month period. DNI, MPXI, C-reactive protein (CRP), and white blood cells (WBCs) were measured in an emergency department and investigated with respect to their abilities to predict the presence of acute complicated appendicitis.

**Results:**

Twenty-nine of the 105 patients (median age, 9 years) were allocated to the complicated group (27.6%) and 76 to the non-complicated group (72.4%). Median serum DNI and CRP were significantly higher in the complicated group [0% vs. 2.2%, p<0.001 and 0.65 mg/dL vs. 8.0 mg/dL, p<0.001], but median MPXI was not (p = 0.316). Area under curve (AUC) for the ability of serum DNI and CRP to predict the presence of acute complicated appendicitis were 0.738 and 0.840, respectively. Multiple logistic regression analyses showed initial CRP [odds ratio 1.301, 95% confidence interval (1.092–1.549), p = 0.003] significantly predicted the presence of a complication. The optimal cutoff for serum CRP was 4.0 mg/dL (sensitivity 69%, specificity 83%, AUC 0.840).

**Conclusions:**

Although serum DNI values were significantly higher in children with acute complicated appendicitis, no evidence was obtained to support the notion that serum DNI or serum MPXI aid the differentiation of acute complicated and non-complicated appendicitis in the ED setting.

## Introduction

Acute appendicitis is the most common cause of abdominal pain encountered in an emergency department setting [1], and the most common illness requiring emergency surgery [2]. Acute appendicitis occurs in almost all age groups, and its delayed diagnosis increases the risk of morbidities, such as, wound infection, abscess formation, or mortality, prolongs hospitalization, and the risk of malpractice litigation. Approximately 30% to 75% of children present with perforation [3,4], and the success of treatment depends on early diagnosis and prompt intervention before complications, such as, perforation, occur [5]. However, in younger children, pain usually manifests as nonspecific signs (irritability, anorexia, lethargy), which are difficult for parents and clinicians to interpret [3], and thus, the identification of complicated appendicitis depends on the results of clinical examination and on elevated inflammatory and biochemical marker levels [6].

Serum delta neutrophil index (DNI) is a new inflammatory marker that provides a measure of the proportion of immature granulocytes in the circulation [7–9]. Several investigators have examined its ability to predict the development of sepsis [10,11], because infectious conditions are known to increase immature granulocyte levels [12,13]. On the other hand, serum myeloperoxidase index (MPXI) is a new inflammatory marker and measure of serum myeloperoxidase (MPO) levels. MPO is released by neutrophils and triggers the synthesis of hypochlorous acid (HOCl) from hydrogen peroxide (H_2_O_2_) and chloride (Cl^−^) [14], and HOCl plays important defensive roles against bacteria, fungi, and viruses [15]. Furthermore, neutrophils extracted from MPO-deficient individuals show lower microbiocidal activities than those extracted from individuals with normal MPO activity [15,16]. We considered serum DNI and MPXI could provide a straightforward means of evaluating inflammation and infection in an emergency department (ED) setting, because the tests required are performed at the same time as routine complete blood count (CBC) testing. However, no information was available on the clinical usefulnesses of serum DNI and MPXI as early predictors of acute complicated appendicitis in children.

Therefore, the aim of this study was to investigate the usefulnesses of serum DNI and MPXI as early predictors of acute complicated appendicitis in children.

## Materials and Methods

### Study design and data

This retrospective observational study was performed on children (<12 years old) with acute appendicitis consecutively treated over a 31-month period (2012 to 2014). The ED department concerned was located in a single urban, tertiary-care hospital (Wonju Severance Christian Hospital, Wonju, Republic of Korea), which has an annual visit volume in excess of 43,000 and is staffed 24 hours per day by board-certified emergency physicians. This study was approved by the institutional review board of Wonju College of Medicine, Yonsei University. Since the study was performed retrospectively and observationally, the patient records and/or information was anonymously processed prior to the analysis.

All patients with the word “appendicitis” in ED discharge codes registered in computerized hospital records were initially considered. For selected patients, diagnosis of acute appendicitis and the presence of a complication were determined based on surgical and pathological findings. Patients with a hematologic abnormality and those who received granulocyte colony stimulating factor, glucocorticoid, or another immunosuppressant (all of which as can affect serum DNI and MPXI) before study enrollment were excluded, as were patients transferred to another hospital, discharged with against medical advice, or not treated surgically.

Data was collected retrospectively by two emergency physicians blinded to the study objectives and hypothesis by reviewing electronic medical records. Disagreements were resolved by consensus. These two reviewers were trained beforehand to reduce bias. The following information was obtained from medical records: age, gender, time from symptom onset to ED arrival, initial vital signs and symptoms, abdominal physical examination findings, inflammatory markers (measured in the ED; including DNI, MPXI, white blood cell (WBC), and C-reactive protein (CRP)), surgical technique, drain insertion, and the presence of complications, such as, perforation, abscess, or peritonitis.

A specific type of automatic cell analyzer (ADVIA 120/2120; Siemens, Tarrytown, NY, USA) was used to determine serum DNI and MPXI values. This is flow cytometry-based hematologic analyzer uses two independent WBC counting methods by using a myeloperoxidase (MPO) channel and a lobularity/nuclear density channel. DNI values were calculated using the following formula: DNI = (leukocyte subfraction assayed using the MPO channel by cytochemical reaction)–(leukocyte subfraction assayed using the nuclear lobularity channel based on reflected light beam measurements) [9,17]. Mean MPXI was calculated using the above-mentioned blood autoanalyzer using 4-chloro-1-naphthol (a substrate for MPO in granulocytes), and black precipitates are formed in these cells. When the stained WBCs pass to the flow cell, light scatter (y-axis) and absorbance (x-axis) are measured using a tungsten-halogen light source, and the deviation from mean of the x-axis values of neutrophils from an archetypal population defines the MPXI [18]. Unlike other biomarkers such as serum CRP that requires additional laboratory processes and costs, serum DNI and MPXI analysis do not require any additional cost or time in the clinical setting, because they are performed routinely with leukocyte differential counts and the results can be obtained at the same time as WBC counts and neutrophil fractions in CBC testing.

Laparoscopic appendectomy was performed using three ports (anti-McBurney’s point, supra-pubic and supra- or infra-umbilical ports). When complicated appendicitis was suspected, in some cases open appendectomy was performed. In cases of perforated appendicitis or with profuse fluid collection, a closed suction drain with its tip placed in the pelvic cavity was inserted via a supra-pubic trocar site.

Patients were allocated to a non-complicated group (simple acute appendicitis) or a complicated group (perforation, abscess, or localized or generalized peritonitis) based on surgical and histological findings [19].

### Statistical analysis

Categorical variables are presented as frequencies and percentages, and continuous variables as means and standard deviations (SD) or as medians and interquartile ranges (IQRs). Normality was assessed using the Shapiro-Wilk. The chi-square test or Fisher’s exact test were used to compare categorical variables, while the two-sample t-test or the Mann-Whitney U-test were used to compare continuous variables. Area under curve (AUC) for predictive ability for the presence of acute complicated appendicitis were determined using receiver operation characteristic (ROC) curves. Variables with p values of <0.05 by univariate analysis were entered into the multiple logistic regression analysis to identify early predictors of acute complicated appendicitis. P-values of <0.05 were considered statistically significant, and the analysis was performed using SPSS Ver. 20 (IBM, Aramark, NY, USA).

## Results

### Characteristics of the study subjects

One hundred and eighteen consecutive acute appendicitis patients were treated during the study period. Some patients were excluded for the following reasons: transfer to another hospital (5 patients), appendicitis not confirmed by surgical findings (5 patients), and insufficient data (3 patients). Therefore, 105 patients were finally included.

The baseline characteristics of the 105 study subjects are shown in Table 1. Sixty-three were boys (60.0%) and the overall median age 9 years. Median time from symptom onset to ED arrival was 18.2 hours. Common initial symptoms at ED presentation were right lower quadrant (RLQ) pain (79.0%) and vomiting (56.2%). RLQ rebound tenderness was observed in 30.5% of patients. Median hospital stay was 4 days.

**Table 1:** Baseline characteristics of children with acute appendicitis.

Characteristics	Total (n = 105)	Non-complicated group	Complicated group	p-value
		(n = 76; 72.4%)	(n = 29; 27.6%)	
Age (years)	9.0 (7.0–11.0)*	9.0 (6.0–10.8)*	9.0 (8.0–11.0)*	0.087
Boy	63 (60.0%)	47 (61.8%)	16 (55.2%)	0.533
Time from symptoms to ED arrival (hours)	18.2 (7.2–31.7)*	13.5 (5.9–24.0)*	35.0 (15.9–60.6)*	<0.001
SBP (mmHg)	117.6±12.0†	116.6±10.7†	120.4±14.9†	0.324
PR (beats/minute)	101.6±18.0†	99.8±18.6†	106.4±15.9†	0.269
BT (°C)	37.1±0.8†	37.1±0.7†	37.3±1.0†	0.075
Symptoms & signs				
Vomiting	59 (56.2%)	40 (52.6%)	19 (65.5%)	0.234
RLQ pain	83 (79.0%)	60 (78.9%)	23 (79.3%)	0.967
RUQ pain	5 (4.8%)	5 (6.6%)	0 (0%)	0.319
Periumbilical pain	29 (27.6%)	17 (22.4%)	12 (41.4%)	0.051
Epigastric pain	14 (13.3%)	11 (14.5%)	3 (10.3%)	0.753
Physical examinations				
RLQ tenderness	92 (87.6%)	67 (88.2%)	25 (86.2%)	0.750
RLQ rebound tenderness	32 (30.5%)	17 (22.4%)	15 (51.7%)	0.003
Inflammatory markers				
DNI (%)	0.1 (0–1.7)*	0 (0–1.1)*	2.2 (0.1–3.9)*	<0.001
MPXI	2.1 (0–4.1)*	2.0 (-0.3–4.4)*	3.0 (0.8–4.0)*	0.316
WBC (cells/mL)	16310 (13425–18875)*	15680 (13368–18843)*	17360 (14175–19575)*	0.333
CRP (mg/dL)	1.91 (0.29–6.19)*	0.65 (0.29–2.80)*	8.0 (2.34–12.75)*	<0.001
Surgical technique				
Laparoscopic surgery	99 (94.3)	72 (94.7)	27 (93.1)	0.531
Open surgery	6 (5.7)	4 (5.3)	2 (6.9)	
Drain insertion	29 (27.6)	10 (13.2%)	19 (65.5%)	<0.001
Total admission days	4 (3–5)*	3 (2–4)*	5 (4–6)*	<0.001

ED, emergency department; SBP, systolic blood pressure; PR, pulse rate; BT, body temperature; RLQ, right lower quadrant; RUQ, right upper quadrant; DNI, delta neutrophil index; MPXI, myeloperoxidase index; WBC, white blood cell; CRP, C-reactive protein

* Median (interquartile range).

† Mean±Standard deviation.

The complicated group included 29 patients (27.6%). Patients in the non-complicated and complicated groups differed significantly in terms of time from symptom onset to ED arrival (13.5 hours vs. 35.0 hours, respectively, p<0.001) and RLQ rebound tenderness (22.4% vs. 51.7%, respectively, p = 0.003). Proportions that underwent open appendectomy were similar in the non-complicated and complicated groups (5.3% vs 6.9%, respectively, p = 0.531). Drain insertion was more common in the complicated group (13.2% vs 65.5%, p<0.001), and median hospital stays in the non-complicated and complicated groups were 3 and 5 days (p<0.001) (Table 1).

**Fig 1:**
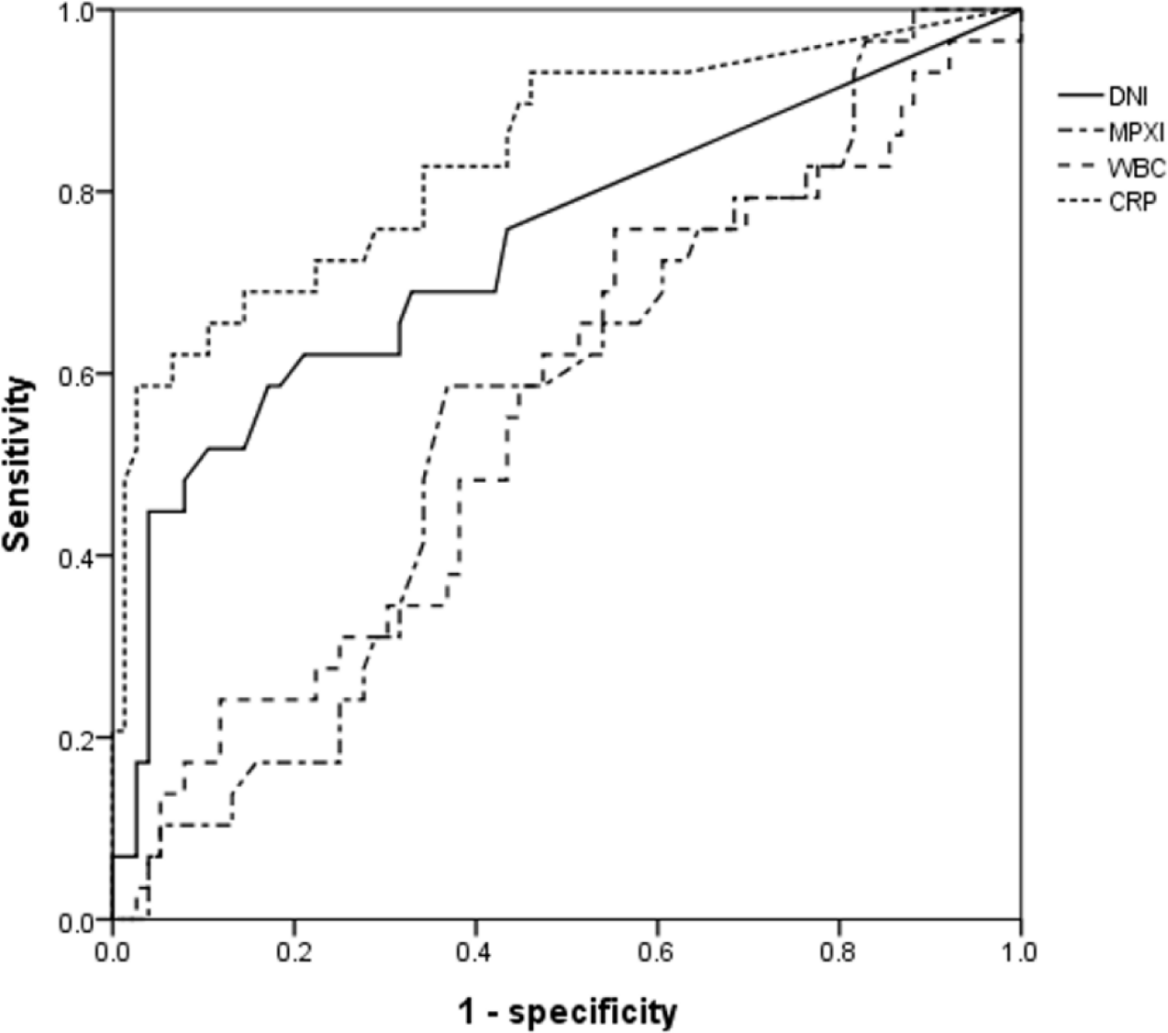
Receiver operating characteristic curves of inflammatory markers. DNI, delta neutrophil index; MPXI, myeloperoxidase index; WBC, white blood cell; CRP, C-reactive protein.

Median serum DNI and CRP were significantly higher in the complicated group (0% vs. 2.2%, p<0.001 and 0.65 mg/dL vs. 8.0 mg/dL, p<0.001), but median serum MPXI and WBC were non-significantly higher in the complicated group (Table 1). AUCs for the ability of serum DNI and CRP to predict the presence of acute complicated appendicitis were 0.738 and 0.840, respectively. Multiple logistic regression analyses showed that initial CRP significantly predicted the presence of complicated appendicitis [odds ratio (OR) 1.301, 95% confidence interval (CI) (1.092–1.549), p = 0.003]. The optimum cutoff for initial serum CRP was 4.0 mg/dL (sensitivity: 69%, specificity: 83%, and AUC 0.840) (Tables 2 and 3, Fig 1).

**Table 2:** AUCs and 95% CIs of inflammatory markers.

Markers	AUC (95% CI)
DNI	0.738 (0.622–0.854)
MPXI	0.564 (0.446–0.681)
WBC	0.561 (0.438–0.685)
CRP	0.840 (0.748–0.932)

AUC, area under the curve; CI, confidence interval; DNI, delta neutrophil index; MPXI, myeloperoxidase index

WBC, white blood cell; CRP, C-reactive protein

**Table 3:** Predictors of acute complicated appendicitis as determined by multivariate logistic regression analysis.

Variables	OR	95% CI	P-value
Time from symptoms to ED arrival (hours)	1.014	0.997–1.030	0.106
RLQ rebound tenderness	2.384	0.735–7.738	0.148
DNI (%)	1.092	0.837–1.425	0.517
CRP (mg/dL)	1.301	1.092–1.549	0.003

ED, emergency department; RLQ, right lower quadrant; DNI, delta neutrophil index; CRP, C-reactive protein; OR, odds ratio; CI, confidence interval

## Discussion

This is the first report on the abilities of serum DNI and MPXI to predict the presence of complications in children with acute appendicitis. We found serum DNI was significantly higher in the complicated group, which is in-line with increases in immature granulocyte proportions in the circulation with disease progression due to bacterial infection of peri-appendiceal structures. However, the predictive value of serum DNI for complicated appendicitis was only fair (AUC 0.738) and lower than that of serum CRP. Nevertheless, we believe a well-designed prospective study is needed to determine the predictive merits of serum DNI, because although its ability to predict the presence of a complication was only fair, DNI values were found to differ significantly between non-complicate group and complicated group, and if DNI does have value, testing could be easily performed in the ED setting.

On the other hand, MPXI was found to be non-significantly higher in the complicated group, and ROC curve analysis confirmed MPXI could not differentiate the two groups (AUC 0.564). In a study by Yonezawa et al. [18], it was reported that although the elevated MPXI indicates increased MPO activities for microbicidal activities in infectious state, because MPXI level is presumably controlled by the process of synthesising and releasing of MPO, in severe bacterial infections such as sepsis, the activated neutrophils release large amounts of MPO for bactericidal activities, and an increase in degranulated neutrophils can reduce the MPXI values. Therefore, we thought that serum MPXI may not increase significantly in septic condition out of complicated group.

Serum CRP is widely used as an objective index of disease activity in the ED setting. CRP is a plasma protein and its concentration increases dramatically as a result of cytokine-mediated responses to most forms of tissue injury, infection, and inflammation [20]. The present study shows serum CRP is a good predictor of complicated appendicitis (Tables 2 and 3). Actually, a number of studies, which included subjects at time of surgery, have indicated CRP is sensitive (83% to >90%) for detecting appendiceal perforation and abscess formation, which are both more commonly found in children [4,21,22]. However, as mentioned above, these biochemical tests should only be used as guides, because considerations of the wider clinical picture are of greater diagnostic importance [23].

In the present study, the overall complication rate for perforation, peritonitis, and abscess was 26.6% (38 patients), and perforation developed in 33 patients (23.1%), which concurs with previous reports of perforation rates at diagnosis of from 17% to 50% [19,24,25]. Furthermore, this finding re-confirms that the complication rate is high in pediatric cases with acute appendicitis.

We found that time from symptom development to ED presentation was significantly greater in the complicated group, which is in-line with expectation, because a delayed diagnosis would allow the condition to progress. In addition, abdominal rebound tenderness was more common in the complicated group, presumably because inflammatory reactions were more severe and peritonitis was more likely.

It should be borne in mind that the present study was limited by its retrospective design, which meant not all data was available, especially data on initial symptoms and physical examinations. Furthermore, because the study was conducted at the emergency center of one hospital and the cohort was relatively small its results should be regarded with caution. Nevertheless, all children treated for acute appendicitis after serum DNI and MPXI kits became commercially available were included in the study to reduce bias. In addition, the impact of serial serum DNI values on outcomes were not investigated, and the usefulnesses of DNI and MPXI for determining response to definitive treatment were not examined.

## Conclusions

Although serum DNI was found to be significantly higher in the complicated group than in the non-complicated group, this study offers no evidence to support the notion that serum DNI or serum MPXI aids the differentiation of acute complicated and acute non-complicated appendicitis in the ED setting.
